# Detection of *Leishmania donovani* DNA from Oral Swab in Visceral Leishmaniasis

**DOI:** 10.3390/pathogens14020144

**Published:** 2025-02-04

**Authors:** Santana R. Sarkar, Rina Hobo, Yuki Shoshi, Shyamal K. Paul, Yasuyuki Goto, Eisei Noiri, Yoshitsugu Matsumoto, Chizu Sanjoba

**Affiliations:** 1 Department of Microbiology, Mymensingh Medical College, Mymensingh 2206, Bangladesh; dr.santanasarkar@gmail.com (S.R.S.); drshyamal10@yahoo.com (S.K.P.); 2 Laboratory of Molecular Immunology, Department of Animal Resource Sciences, Graduate School of Agricultural and Life Sciences, The University of Tokyo, Tokyo 113-8657, Japanyshoshi@g.ecc.u-tokyo.ac.jp (Y.S.); aygoto@g.ecc.u-tokyo.ac.jp (Y.G.);; 3 Hemodialysis and Apheresis, Nephrology 107 Lab, The University of Tokyo Hospital, Tokyo 113-8655, Japan; noiri-tky@umin.ac.jp

**Keywords:** Bangladesh, *Leishmania donovani*, oral swab, visceral leishmaniasis

## Abstract

Visceral leishmaniasis (VL) is the most severe form of leishmaniasis and is fatal if left untreated in over 95% of cases. Leishmaniasis is one of the neglected tropical diseases that tend to thrive in developing regions of the world where inadequate access to healthcare makes it difficult for some people to even receive a diagnosis. This study examined the usefulness of oral swabs as specimens for VL diagnosis, by detecting *Leishmania donovani* DNA in oral swabs from both VL patients and *L. donovani*-infected mice. Eighty oral swab (OS) and blood buffy coat (BC) samples were collected from suspected VL cases in Bangladesh. These samples were evaluated using *Leishmania kinetoplast minicircle DNA* (*kDNA*) in real-time PCR, and the results showed that 62.5% (50/80) and 67.5% (54/80) of the cases tested positive for the BC specimen and OS, respectively. The OS positivity was statistically comparable to the BC. *L. donovani* DNA was also detected in an oral swab of all infected BALB/c mice by conventional PCR targeting the *large subunit ribosomal RNA gene* (*LSUrRNA*), while it was negative in uninfected mice. This study highlights the potential of practical methods for the molecular diagnosis of VL using oral swabs as a non-invasive, simple, and accurate approach.

## 1. Introduction

Visceral leishmaniasis (VL), also known as kala-azar, is the most severe form of human leishmaniasis in which *Leishmania* parasites migrate to vital organs like the liver, spleen, and bone marrow [1]. Patients typically exhibit irregular episodes of fever, weight loss, splenomegaly, hepatomegaly, and anemia. If left untreated, this condition can be fatal [2]. It estimates that 50,000 to 90,000 new cases of VL occur annually, with 5000 to 10,000 deaths each year [2]. In the Indian subcontinent, approximately 200 million people are estimated to be at risk of developing VL, accounting for about 67% of the global disease burden of VL. Despite significant progress in reducing the number of cases, certain areas remain highly endemic, and new foci of transmission have emerged, indicating the need for continuous vigilance and intervention [3]. In spite of the high endemicity, a large number of VL cases are not reported for many reasons, such as the lack of a proper surveillance system, healthcare access barriers, and lack of awareness [4,5]. In recent years, Bangladesh has achieved significant progress in reducing the burden of kala-azar, particularly among socially marginalized and impoverished communities. The incidence of the disease has been consistently declining, reaching a milestone of less than 1 case per 10,000 people (elimination level) [6,7]. In this scenario, an accurate diagnosis is crucial for detecting *Leishmania* infection and identifying the initial signs of a potential re-epidemic. Timely control measures can then be implemented to effectively manage the situation.

The molecular diagnosis of VL using PCR is a reliable and valuable method for detecting *Leishmania* infection in aspirates from the spleen, lymph nodes, bone marrow, and buffy coat. This technique is considered highly effective in accurately diagnosing VL [8]. However, invasive sampling methods are difficult to perform in settings with limited resources and training. Additionally, biopsies are often delicate, uncomfortable, and painful for patients with severe illness or elderly people, babies, and people who are unwilling to be subjected to this invasive procedure. Moreover, while taking tissues, bleeding may result at the biopsy sites [9,10]. Oral fluid is becoming ever more popular as a sample type for non-invasive diagnostic techniques. Diagnosis testing with oral fluid has been validated for detecting antivirus antibodies [11,12], and its usefulness in diagnosing malaria, the protozoan infection, has also been reported [13,14]. Although diagnosis using oral swabs has been evaluated in several studies in VL patients [9,10,15,16], and in canine leishmaniasis [17,18], this is not a sufficient empirical example to standardize it as a diagnostic method for leishmaniasis. This is because of the small number of cases or different types of *Leishmania*. This emphasizes the necessity of further study on the diagnostic value of saliva samples for VL.

An oral swab is a cost-effective and safe technique to isolate DNA for various biological experiments particularly large epidemiological studies [19]. Non-invasive samples with oral swabs can be collected in rural areas, transported without temperature considerations, and referred to central laboratories for testing through PCR to ensure efficient and accurate diagnosis. Utilizing non-invasive sampling techniques along with sensitive and straightforward molecular diagnostics can serve as an asset in addressing the challenges associated with controlling VL. Moreover, swab samples afford larger flexibility for storage, transportation, or long-time preservation [20]. Additionally, it is important to determine whether the amount of *L. donovani* DNA detected in an oral swab corresponds with the level of parasite load. *L. donovani* DNA detection in oral swabs may be used as an efficient treatment assessment by proving the link because of how simple and non-invasive the collection process is. The mouse model frequently enables the comparison of *L. donovani* DNA quantities obtained from oral swabs with the number of *L. donovani* parasites present in organ samples at various infection stages [21]. This study highlights the utilization of molecular techniques to extract oral cells from swab samples for the purpose of diagnosing VL using *Leishmania* DNA. Thus, by comparing with the mouse model, the detection system appears to be valid and accurate. This research also demonstrated that mouth swabs from infected mice could contain *L. donovani* DNA. This is the first study on the presence of *L. donovani* DNA in oral swab samples from VL-infected individuals in Bangladesh.

## 2. Materials and Methods

### 2.1. Selection of Cases for Specimens

A total of 80 Kalazar Detect™ Rapid Test for Visceral Leishmaniasis (rk-39 test: InBios International, Seattle, WA, USA) positive VL cases were selected during the period of February 2016 to January 2017 from Surya Kanta Kala-azar Research Center (SKKRC), Mymensingh region of Bangladesh (24.5212° N, 90.4124° E). VL was suspected when a patient came to the hospital from a VL endemic area and had a history of fever for more than two weeks, weight loss, and a palpable spleen in most cases. Serum samples of VL-suspected patients were submitted to rk-39 test, the strip test widely used for the diagnosis in endemic countries, and positive patients were included in this study. Different clinical forms of VL were enrolled in the study. Individuals who were diagnosed with VL by positive rk-39 test with symptoms and no history of treatment for VL before (Primary Kala-azar: PKA) along with second and subsequent VL and history of treatment for VL (Recurrent Kala-azar: RKA) were considered as VL cases [8] (Table 1). RKA patients were treated with liposomal amphotericin B and/or miltefosine, following national guidelines. Sampling was conducted before treatment started. No significant concomitant infections or diseases were identified in RKA cases, though factors like immune suppression and malnutrition remain relevant.

Informed consent from each of the cases was obtained before taking blood and oral swab samples. The spleen and liver were measured using ultrasound scans (Hitachi-Aloka, Tokyo, Japan) in addition to the history and clinical examination. All types of probable VL (PKA+RKA) cases, as well as 10 post kala-azar dermal leishmaniasis (PKDL) cases and 10 healthy participants from endemic and non-endemic VL areas in Bangladesh, were enrolled. To focus on the clinical and diagnostic specifics of PKA and RKA cases, the PKDL patients (*n* = 10) and healthy volunteers (*n* = 10) were not included in the analysis. It is important to note that this study did not include HIV testing. The research protocol was approved by the Ethical Review Committee of Mymensingh Medical College.

### 2.2. Blood Sample Collection and DNA Extraction

In total, 5 mL of venous blood was collected from each participant into a sterile screw-capped plastic tubes containing EDTA anticoagulant for buffy coat PCR and complete blood count (CBC) and erythrocyte sedimentation rate (ESR). For each biomarker, a groupwise median ± Interquartile range was expressed and compared using the Mann–Whitney U test. Significance was defined as *p* < 0.05. The blood sample was centrifuged at 3000× *g* for 5 min to separate the buffy coat. According to the manufacturer’s instructions, DNA was extracted from a 200 µL buffy coat using a QIAamp DNA blood and tissue mini kit (QIAGEN, Hilden, Germany). Purified DNA samples were preserved at −20 °C until use, and the extracted DNA was used to perform real-time PCR using the Applied Biosystems™ Step One Plus™ real-time PCR system, as described earlier by Hamasaki et al. 2016 [22].

### 2.3. Swab Sample Collection and DNA Extraction

Oral swab samples were collected from all the participants in the morning, before they brushed their teeth and right after waking up. Swabs were taken from the mouth specifically from the inner surface of both cheeks by rubbing and rotating the swab 15 to 20 times, applying firm pressure (slightly uncomfortable but not painful to the participants). It was ensured that as much of the swab’s surface was in contact with the cheek’s lining as possible [23]. The swab samples were immersed in 50 µL sterile phosphate-buffered saline solution in a falcon tube. The DNA extraction of oral swab samples was performed using the DNeasy^®^ Blood and Tissue Kit (QIAGEN, Hilden, Germany), according to the manufacturer’s recommendations. The DNA was eluted by 50 µL of buffer AE, and then filtrated and stored at −20 °C.

### 2.4. Multiplex Real-Time PCR

The real-time PCR method was performed according to the methodology described by Hamasaki et al. [22] to detect *Leishmania donovani* DNA, targeting the cysteine protease B gene. Mammalian ribonuclease P RNA component H1 (RPPH1) was used as the endogenous control for real-time PCR quantification of the clinical sample (Assay set no. CCU001S, CCU001SNR, Thermo Fisher Scientific, Waltham, MA, USA). The dye labels used were FAM, VIC, and ABY, and the probe types were MGB, QSY, and QSY. The real-time PCR method was performed in a final total volume of 20 µL containing 10 µL of Taq Path™ qPCR Master Mix, CG (Thermo Fisher Scientific, Waltham, MA, USA). The final concentrations for the set of primers and probes were optimized as follows: 900 nM of *kDNA* primers, 250 nM of *RPPHI* primers, 250 nM of probes to detect *kDNA*, and 150 nM of probes to detect *RPPHI*. The amount of DNA samples for each reaction was 1.5 µL patient DNA. A Step One Plus real-time PCR system (Thermo Fisher Scientific, Waltham, MA, USA) was used as the real-time PCR system.

### 2.5. Leishmania donovani Infection Model in Mice

Male BALB/cA mice (Japan Clea, Tokyo, Japan), aged 6–8 weeks, were used for experiments. The mice were maintained under specific pathogen-free conditions, and all animal experiments were reviewed and approved by the Animal Experiment Committee at the University of Tokyo (approval no p14-930). *Leishmania donovani* D10 (MHOM/NP/03/010) was obtained from the Institute of Tropical Medicine Nagasaki University (NBRP) in Nagasaki, Japan [24]. Promastigotes of *L. donovani* were cultured in TC199 medium (Nissui Pharmaceutical, Tokyo, Japan) together with 25 mM HEPES buffer (MP Biomedicals, Illkirch-Graffenstaden, France) and 10% heat-inactivated fetal bovine serum (Thermo Fisher Scientific, Waltham, MA, USA). All studies were conducted with promastigotes in the stationary phase. According to a previous description [25], the stationary phase of l × 10^8^ *L. donovani* Dl0 promastigotes was inoculated intraperitoneally to BALB/cA mice to simulate an infection. At 15 weeks post-infection, BALB/cA mice were euthanized.

### 2.6. Oral Swab Sampling from Mice and DNA Extraction

The oral swab was obtained from *L. donovani*-infected BALB/cA mice at 12 weeks post-infection. Age-matched BALB/cA mice without infection had oral swabs obtained as controls. Oral swab samples were collected using Nylon^®^ sterile swabs (Copan, Brescia, Italy). A swab was firmly rubbed against the oral cavity and immersed in 500 µL of sterile phosphate-buffered saline. The DNeasy^®^ Blood and Tissue Kit (QIAGEN, Hilden, Germany) was used to extract the DNA from oral swab samples according to the instructions provided by the manufacturer. By using 20 µL of buffer AE, the DNA was eluted.

### 2.7. Detection of Leishmania DNA by LSU rRNA Gene from Oral Swab of L. donovani-Infected BALB/cA Mice

One target gene, *large subunit ribosomal RNA (LSUrRNA)*, was used to find *Leishmania* parasite DNA. *L. donovani* HU3 (MHOM/ET/67/HU3) nucleotide sequences for the *LSU rRNA* gene were aligned. The designed primers were as follows: *LSUrRNA*-forward (5′-GGC GGG CAA CGA AGT GCA AGA AT-3′) and *LSUrRNA*-reverse (5′-GCA CAC TCC AAC GCA ACC CAC GG-3′). The following 25 µL PCR mixture was used for the PCR of *the LSU rRNA* gene: 10×PCR buffer, 2 mM dNTPs, 25 mM MgCl_2_, 1.25 U Taq DNA polymerase, and 2 µL DNA. PCR was carried out for 35 cycles. Cycle conditions were 5 min at 95 °C, 30 s at 95 °C, 30 s for 60 °C, and 30 s for 72 °C. The final cycle was extended by 10 min at 72 °C. *Cytochrome b* (*cytb*) was designed for the detection of mammal DNA. The designed primers were as follows: *cytb*-L14841 (5′-AAA AAG CTT CCA TCC AAC ATC TCA GCA TOA TGA AA-3′) and *cytb* H15149 (5′-AAA CTG CAG CCC CTC AGA ATG ATA TIT GTC CTC A-3′).

### 2.8. Sequencing of the LSU rRNA Gene

PCR was conducted for 35 cycles using 4 µL of genomic DNA as the template and 25 pmol of the *LSUrRNA* primers: *LSUrRNA*-forward (5′-GGC GOG CAA CGA AGT GCA AGA AT-3′) and *LSUrRNA*-reverse (5′-GCA CAC TCC AAC GCA ACC CAC GG-3′) as mentioned above, 0.3 mM of each dNTP, l mM MgSO_4_, 0.3 µM of each primer mix, 1-unit Plastinum Pfx DNA Polymerase, 1×–2× Pfx Amplification Buffer recommended by the manufacturer. Cycling conditions were 5 min at 94 °C, 15 s at 94 °C, 30 s at 60 °C, and 30 s at 68 °C. An additional 5 min at 68 °C was added to the final cycle. After amplification, the products were size selected from agarose gels and purified by a QIAquick Gel Extraction Kit (250) (QIAGEN, Hilden, Germany). The Applied Biosystems 3730xl DNA analyzer was used to determine the nucleotide sequences (Thermo Fisher Scientific, Waltham, MA, USA). The nucleotide sequences were analyzed by DNASIS-MAC (Hitachi Software Engineering Co., Ltd., Yokohama, Japan).

### 2.9. Determination of Spleen and Liver Parasite Burden of L. donovani-Infected BALB/cA Mice

To assess organ weight and determine the parasite burden, the liver and spleen were harvested from the infected mice. Giemsa-stained multiple impression smears of the spleen and liver were obtained. Organ parasite burden, expressed as Leishman–Donovan unit (LDU), was calculated as the number of amastigotes/1000 host cell nuclei × organ weight (mg) [25].

### 2.10. Statistical Analysis

The results of qPCR positivity in two different methods for specimen preparation, oral swab and buffy coat, were examined by calculating kappa values (κ). The agreement was graded by kappa value as fair (0.2–0.4), moderate (0.41–0.6), substantial (0.61–0.8) or great (>0.81). Swab PCR positivity and symptoms were compared using Fisher’s Exact Test. Significance was defined as *p* < 0.05. Calculating the kappa value and Fisher’s Exact Test was conducted by R for Windows (R 4.2.0, R Core Team). For spleen size measured by ultrasound scanning and each biomarker, a groupwise median ± Interquartile range was expressed and compared using the Mann–Whitney *U* test by GraphPad Prism 9 software (GraphPad Software, Inc., San Diego, CA, USA). Significance was defined as *p* < 0.05.

## 3. Results

### 3.1. DNA Detection from Oral Swab and Buffy Coat Specimens by Real-Time PCR

Among the 80 suspected VL cases, 67.5% (54/80) of the oral swab (OS) samples and 62.5% (50/80) of the buffy coat (BC) samples tested positive by real-time PCR targeting both *kDNA* and *cpB*. Forty-six (57.5%) of the cases tested positive for both BC PCR and OS PCR, while 27.5% of the cases tested negative for both tests. Two methods of specimen preparation showed acceptable concordance (κ = 0.671) (Table 2). OS/BC samples of 10 PKDL patients were all negative. In total, 10 healthy volunteers in Bangladesh were also negative in both OS/BC PCR. All OS specimens were confirmed for the presence of human DNA by real-time PCR targeting RPPHI. The positive rate of OS samples from PKA is 62.9% (34/47), which is higher than the positive rate of OS samples from RKA at 37.1% (20/33).

### 3.2. Relation of Oral Swab PCR-Positive Cases with Their Symptoms of the Disease

In total, 52 patients (67.5%) and 24 patients (34.8%) had palpable spleen and liver, respectively. Fever lasting more than 1 month was defined as prolonged fever. The majority of the patients (70%) showed prolonged fever, suggesting a long period had passed after onset of VL. Splenomegaly showed a relationship with OS PCR positivity (*p* < 0.001), while hepatomegaly and fever length did not (Table 3). A high positivity rate (86.5%) was found in patients with palpable spleen. The level of splenomegaly found by physical examination and confirmed by ultrasound was also associated with the OS PCR-positive results (*p* < 0.05). Age categories, sex, and type of disease (PKA or RKA) did not show any significant relation to the result of OS PCR (Table 1).

### 3.3. Relation of Oral Swab PCR-Positive Cases with Their Hematological Parameters

**Figure 1:**
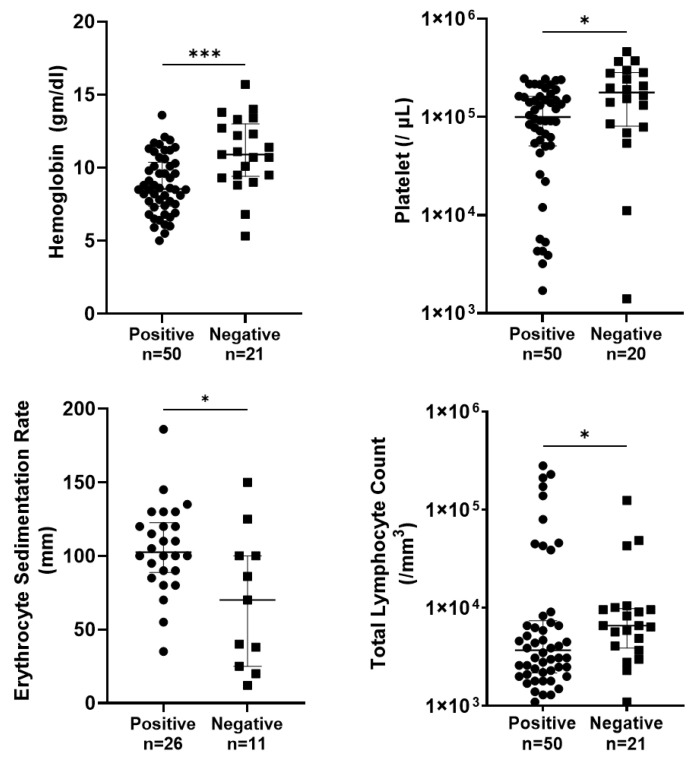
Levels of biomarkers (hemoglobin, platelet, total leucocyte count (TLC), erythrocyte sedimentation rate) were shown within the oral swab PCR-positive group and PCR-negative group. Groups were compared using the Mann–Whitney U test. * *p* < 0.05, *** *p* < 0.001. Error bars show median ± Interquartile range.

Blood count examination results of the oral swab (OS) PCR-positive and -negative cases are shown in Figure 1. It has been shown that a hemoglobin (Hb) level less than 10 g/dL indicates anemia, platelet count less than 150,000 cell/mm^3^ indicates thrombocytopenia, and total leucocyte count (TLC) less than 3300 cells/mm^3^ and erythrocyte sedimentation rate (ESR) more than 20 mm in the first hour indicates an abnormality in blood examination of the cases. It was observed that the levels of hemoglobin and platelets were significantly lower in OS PCR-positive cases (*p* < 0.001 and *p* < 0.05, respectively). Total leucocyte count (TLC) count was also significantly decreased in OS-positive cases (*p* < 0.05). The ESR level was significantly increased in OS-positive cases (*p* < 0.05).

### 3.4. Parasitism and Molecular Detection in BALB/cA Mice Infected with L. donovani

**Figure 2:**
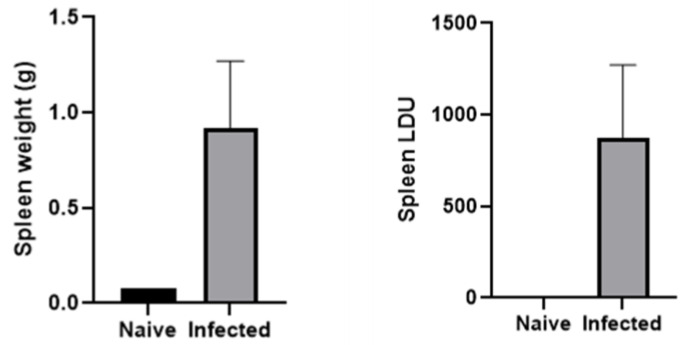
Spleen weight of *L. donovani*-infected (*n* = 5) and naïve mice (*n* = 1) at 15 weeks p.i. Parasite burden of spleen of infected (*n* = 5) and naïve mice (*n* = 1) at 15 weeks p.i. Leishman–Donovan units (LDUs), the number of amastigotes per 1000 host nucleated cells x organ weight (g).

At 15 weeks post-inoculation (p.i.), *L. donovani*-infected BALB/cA mice exhibited significant parasitism in the spleen. To assess the infection, the Leishman–Donovan units (LDUs) were calculated, and both spleen weight and parasite loads were evaluated (Figure 2). The mean spleen weight in infected mice at 15 weeks (*n* = 5) was 918.2 ± 352.4 mg, with an LDU of 870.1 ± 401.3, whereas naïve mice weighed 77 mg. The spleen weight of *L donovani*-infected mice was ten times greater than that of naïve mice.

**Figure 3:**
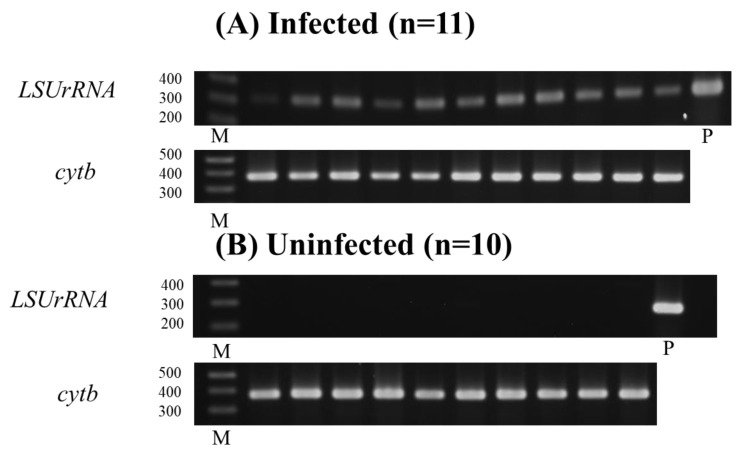
(**A,B**) PCR for *LSUrRNA* was positive in all infected mouse samples (311 bp) (**A**), but negative in all uninfected mouse samples (**B**) PCR for *cytb* was positive in all samples (374 bp), confirming the presence of mouse DNA. M: 1 kb Plus Ladder, P: 20 ng *L. donovani* D10 DNA.

To further investigate the presence of *L. donovani*, conventional PCR targeting the *LSUrRNA* gene was performed on oral swab samples collected at 12 weeks p.i. BALB/cA mice (*n* = 11) and uninfected mice (*n* = 10) were analyzed to see if *L. donovani* parasite DNA was present (311 bp). All infected mice (*n* = 11) displayed a band of approximately 311 bp, corresponding to *L. donovani* DNA, while no such bands were observed in uninfected mice. *Cytochrome b* (*cytb*) PCR confirmed the presence of mouse DNA in all samples, yielding positive results (374 bp) (Figure 3A,B).

The PCR-positive samples were subsequently subjected to *LSUrRNA* gene sequencing, which confirmed the complete identity of the oral swab DNA from infected mice with the *L. donovani* parasite DNA, thus validating the PCR findings.

## 4. Discussion

Non-invasive methods for collecting biospecimens are invaluable for diagnostics, clinical trials, and epidemiological screening studies, particularly in remote areas and among children. In regions like Bangladesh, where disease prevalence is decreasing, people often hesitate to visit health centers for traditional blood-based VL screening. In such contexts, collecting oral swab samples non-invasively from remote locations and transporting them to laboratories offers a practical solution for confirming VL diagnosis. This approach not only enhances accessibility to diagnostic services but also supports effective disease surveillance and management strategies in low-burden settings.

The demographic profile of our VL patient population aligns with previous studies conducted in endemic regions of Bangladesh, with the predominant age group being 15–45 years, constituting 57.5% of cases [26]. This age group typically represents individuals most actively involved in agricultural and occupational activities, which may increase their exposure to the sandfly vector. The role of sex in VL susceptibility has been a subject of conflicting findings in the literature. While some studies have reported a higher risk among male participants [27,28], others, including those conducted in Bangladesh and India, have not observed a significant association between sex and VL incidence [26,29]. Our study corroborates the latter findings, demonstrating an equal distribution of cases between males and females. In our study, the even distribution between males and females suggests that both genders are equally at risk, and public health interventions should address both groups equally. The high positivity rate (62.9%) for PKA compared to RKA (37.1%) indicates that individuals with PKA may have a higher burden of the pathogen or its genetic material, which would make it easier for PCR to detect.

Molecular tests have been proposed as sensitive tools for diagnosing VL [30]. PCR using more intrusive sampling methods, such as the use of buffy coat samples, has been described frequently with variable sensitivities and specificities [31,32]. In our study, we used the buffy coat as a comparator, as it provides a reasonable standard for evaluating oral swabs. Additionally, it is a less invasive method commonly used in resource-limited settings. We detected 67.5% of OS samples positive by real-time PCR. This study corresponds with Pandey et al. [33], who reported a 71% PCR positivity rate of VL in Nepal. To the best of our knowledge, this study is the first to report the presence of *Leishmania donovani* DNA in OS samples of VL infection in Bangladesh.

It is easy to perform and interpret, so it could be used as a screening test in developing areas and among suspected populations [17]. These oral fluids have already been validated for the detection of *Helicobacter pylori* DNA in the saliva of infected individuals [34,35]. Real-time PCR can also be employed with significant precision to predict the cure of the disease. Though the mechanism of *Leishmania* DNA presence in oral swabs is unidentified, in response to reports of parasite presence in bodily fluids such as saliva and urine of asymptomatic individuals, a few studies have suggested the presence of parasite cell-free DNA, or DNA fragments, in the circulation [36,37]. Parasite cell-free DNA (cfDNA) can be released into various host fluid compartments through active secretion and passive mechanisms such as parasite degradation, cellular apoptosis/necrosis, or excretory–secretory products. While recent cancer research has highlighted the diagnostic potential of double-stranded DNA in tumor-derived exosomes [38], the role of exosomes in Leishmania-related cfDNA release remains unclear. Further investigation into the mechanisms of cfDNA release and its potential for VL diagnosis using oral swabs is warranted.

In places where facilities and sanitation are inadequate, using an oral swab to collect samples can be helpful. VL primarily affects the poor in inaccessible rural places [39]. According to a survey performed in rural India, some VL patients died before being diagnosed [40]. In addition, subjects in this location may hesitate or be reluctant to give their blood. Furthermore, blood collection in insufficient settings has the potential to spread infection. Thus, one of the diagnoses for VL could be the detection of *Leishmania* DNA using oral swab collection, which is a non-invasive, simple, and efficient solution.

The study observed the relationship between oral swab PCR-positive cases and fever, spleen, and liver conditions. Among the cases, 52 (67.5%) had palpable spleen, and 24 (34.8%) had palpable liver. The data revealed a significant link between spleen palpability and OS PCR positivity (*p* < 0.001). This strong correlation indicates that spleen palpability is a reliable indicator of VL and is associated with a higher likelihood of detecting Leishmania DNA in oral swabs. This finding aligns with previous research that has identified splenomegaly as a common and crucial clinical feature of VL, often utilized in its diagnosis and prognosis [39]. Our results indicate that the greater positivity rate (86.5%) in patients with palpable spleens, a sign of advanced VL, suggests that OS PCR sensitivity rises with clinical development. This suggests that OS PCR might be a better method for identifying symptomatic and advanced cases than for identifying infections that are early or subclinical. In contrast, no significant association was found between liver palpability and OS PCR positivity. While hepatomegaly is a recognized symptom of VL, its diagnostic value appears to be less significant compared to splenomegaly, as indicated by the current data and existing literature [41]. Similarly, the duration of fever did not show a significant association with OS PCR positivity. Although fever is a common symptom of VL, it is nonspecific and can be linked to various other infections and conditions. Therefore, while fever is an important clinical feature, it does not significantly improve the predictive value of OS PCR for diagnosing VL [42]. The study emphasizes spleen palpability as a vital diagnostic tool for VL, with liver palpability and fever duration proving less reliable on their own. In settings with limited resources, where invasive procedures are less feasible, combining physical examination findings, such as spleen palpability, with non-invasive diagnostic methods like OS PCR could enhance the accuracy and efficiency of VL diagnosis. This approach would be particularly advantageous in endemic areas, facilitating early detection and prompt treatment of the disease.

The blood count examination results, as depicted in Figure 2, highlight significant differences between oral swab (OS) PCR-positive and OS PCR-negative cases of VL. These differences underscore the systemic impact of VL on each parameter, providing valuable insights into the diagnostic and prognostic implications of these findings. The study reveals that hemoglobin levels were significantly lower in OS PCR-positive cases (*p* < 0.0001). Anemia, indicated by Hb levels less than 10 g/dL, is a common manifestation in VL patients and reflects the chronic nature of the disease and its impact on bone marrow function. Earlier scientists have also reported decreased levels of hemoglobin, platelets, and TLC in cases of VL, caused by factors such as hemolysis, sequestration, and suppression of erythropoiesis due to the infiltration of the bone marrow by *Leishmania* parasites [43,44]. Anemia, thrombocytopenia, leukopenia, and elevated ESR are critical markers that not only aid in the diagnosis of VL but also help monitor disease progression and treatment response. Furthermore, anemia, a common clinical feature in VL patients, was frequently observed in OS PCR-positive cases, suggesting its potential as an additional indicator of disease severity and progression. These findings highlight the need for integrated diagnostic approaches combining molecular methods like OS PCR with hematological assessments to improve the detection and management of VL.

By using an experimental mouse, we also measured the parasitism levels at various stages of infection. As expected, the parasite burden in the spleens of infected mice increased as the infection progressed, demonstrating higher levels of parasitism compared to earlier stages. In addition, to investigate the potential for oral transmission of *L. donovani*, *LSU rRNA* gene sequencing was performed on DNA extracted from oral swabs of BALB/cA mice infected for 12 weeks and compared to *L. donovani* parasite DNA. Notably, a perfect match of 100% (207/207 bp) was observed between the *LSU rRNA* gene sequences from these two sources. This finding substantiates the hypothesis that *L. donovani*-derived DNA can be detected in oral swabs, supporting the potential role of saliva during infection. These findings may not directly apply to human disease. However, they help us elucidate the mechanisms by which parasite-derived DNA appears in the oral cavity of animal models. This knowledge can guide further research on transmission routes and possible interventions.

Bangladesh’s current guidelines rely on the rk-39 test to detect suspected VL cases, but this test cannot differentiate between active, past, or subclinical infections. Besides this, in endemic places, 10–20% of healthy individuals test positive for this infection. Antibody detection techniques, including immunochromatography, are commonly used for VL diagnosis, but their sensitivity can vary depending on the patient’s immunological status. The PCR method, on the other hand, directly detects Leishmania DNA, giving more specificity and the ability to identify cases with low antibody titers, like those seen in people with weakened immune systems. Comparing the results obtained with oral swab PCR with those obtained with anti-Leishmania antibodies in saliva and blood would further validate the usefulness of using oral swabs for VL diagnosis. In our study, the OS showed a positive rate compared to the BC specimen, and it could be a promising non-invasive diagnostic option. The relatively low sensitivity of the oral swab PCR for VL, at 67.5%, is a significant limitation of this study. This suggests that a considerable proportion of active infections might have been missed. While PCR offers a more specific diagnostic tool compared to the rK-39 test, the possibility of false-positive results cannot be excluded. Further research is warranted to investigate factors contributing to the low sensitivity of the oral swab PCR, such as low parasite load or inhibitory substances in oral samples. Additionally, it is crucial to optimize the use of PCR results, clinical manifestations, physical findings, and other diagnostic markers and improve the diagnostic algorithm for VL. This report will serve such a purpose.

## 5. Conclusions

Analyzing the findings of this study provides a valid method for diagnosing VL. The study conclusively demonstrates that non-invasive oral swabs are effective samples for molecular diagnosis of VL using real-time PCR technology. However, it was observed that this method may not be as effective for diagnosing post kala-azar dermal leishmaniasis (PKDL) infections. Real-time PCR remains a valuable tool for VL screening and control efforts, providing a practical approach to improve diagnostic accuracy and guide targeted interventions.

## Figures and Tables

**Table 1:** Characteristics of 80 individuals who provided specimens for VL.

		Total (%)	Age			Sex	
			<15	15–45	45<	Male	Female
Type of disease	PKA	47 (58.8)	8	27	12	24	23
	RKA	33 (41.3)	1	19	13	16	17
Total		80 (100)	9	46	25	40	40

**Table 2:** Distribution of real-time PCR results (*n* = 80).

	qPCR		No. of Samples
	OS	BC	
	+	+	46
	+	−	8
	−	+	4
	−	−	22
Positivity rate (%)	67.5	62.5	
	(54/80)	(50/80)	

OS: oral swab samples; BC: blood buffy coat; kappa value (κ) for OS and BC was 0.671 (substantial agreement).

**Table 3:** Relationship between oral swab PCR positivity and symptoms of the disease.

Symptoms of the Disease		Number of Patient (%)	Result of OS PCR		Significance
			Positive (%)	Negative (%)	
Spleen	Palpable	52 (67.5)	45 (86.5)	7 (13.5)	*p* < 0.001
(*n* = 77)	Not palpable	25 (32.5)	9 (36)	16 (64)	
Liver	Palpable	24 (34.8)	20 (83.3)	4 (16.7)	NS
(*n* = 69)	Not palpable	45 (65.2)	30 (66.7)	15 (33.3)	
Fever	Within 1 month	24 (30)	15 (62.5)	9 (37.5)	NS
(*n* = 80)	>1 month	56 (70)	39 (69.6)	17 (30.4)	

NS: not significant.

## Data Availability

The original contributions presented in this study are included in the article. Further inquiries can be directed to the corresponding author.
